# Epigenetic regulation in type II diabetes: linking molecular mechanisms to clinical management

**DOI:** 10.1007/s40200-025-01831-1

**Published:** 2026-02-25

**Authors:** Maryam Chaudhry, Said Sif

**Affiliations:** 1 College of Medicine, Qatar University, Doha, Qatar; 2 Department of Biological and Environmental Sciences, Qatar University, Doha, Qatar

## Abstract

Type II diabetes mellitus (T2DM) is a multidimensional metabolic disorder driven by insulin resistance, chronic inflammation and β-cell dysfunction. Emerging evidence shows that epigenetic mechanisms i.e., DNA methylation, histone acetylation and noncoding RNAs, form a key nexus between genetic predisposition and environmental factors including diet, oxidative stress and obesity. These inheritable yet reversible modifications shape transcriptional control of key genes involved in inflammatory signalling, glucose metabolism and insulin secretion. Altered methylation of PDX1 and GLP-1R genes, overexpression of histone deacetylases, impairment of miRNA expression (e.g., miR-21, miR-146a) and lncRNAs (e.g., MALAT) cumulatively impair insulin sensitivity and β-cell identity. In addition, transgenerational epigenetic inheritance reveals how parental nutrition choices and metabolic status can predispose offspring to metabolic memory of T2DM risk. Emerging evidence highlights the promise of targeting epigenetic modifiers e.g., DNMT, HDAC inhibitors and miRNA-based strategies to reverse the abnormalities and regain normal gene expression and metabolic balance. Complementarily, the Wnt/ β-catenin and GLP-1 signalling pathways are key interfaces of epigenetic modulation of β-cell function. Understanding these mechanisms is a gateway for precision medicine that goes beyond glycaemic control in the direction of disease modification and prevention. Integrating epigenetic profiling into clinical management can redefine patients care therapies covering both molecular and heritable dimensions of T2DM.

**Contents**

- Symptoms and complications of diabetes mellitus
- Epigenetics of type 2 Diabetes Mellitus
  - DNA methylation and gene regulation
  - Histone Modifications And β-cell Dysfunction
  - Noncoding RNAs and Insulin Resistance
- Epigenetics of β-Cell dysfunction and insulin resistance
  - β-Cell Dysfunction and dedifferentiation
- Transgenerational Epigenetics Inheritance in T2DM
- Clinical Implications and Epigenetic Therapies
  - DNA methylation (DNMT) inhibitors
  - Histone Deacetylase (HDAC) Inhibitors
  - microRNA (miRNA)-Based Therapies
  - Ongoing Clinical Trials and Personalised Medicine
- Wnt/β-Catenin Signalling and Epigenetics in T2DM
  - Wnt Signalling and Epigenetic Regulation
  - Wnt/β-Catenin and GLP-1 Receptor Expression
- GLP-1 and Its Epigenetic Regulation in T2DM
  - GLP-1 Biology and Signalling Pathway
  - Epigenetic Regulation of GLP-1 Pathway
- GLP-1 Receptor Agonists (GLP-1RAs) in T2DM Therapy
  - Classification of GLP-1Ras
  - Mechanisms of GLP-1RAs
  - Clinical Benefits of GLP-1Ras
  - Limitations and Side Effects
- Conclusion: Epigenetics as a Target for T2DM Therapy
- Bibliography

Diabetes mellitus is a chronic metabolic disorder characterised by persistent hyperglycaemia due to defects in insulin secretion and or action. It is mainly categorised in Type I and Type II, each with its unique mechanisms and clinical implications [1]. Type I diabetes is primarily an autoimmune condition in which antibodies such as anti-GAD and anti-islet antibodies produced by body’s immune system lead to destruction of pancreatic β-cells. Thus, this reduces insulin production and impairs glucose uptake by cells and resulting in hyperglycaemia. Type I mainly presents in early ages, childhood or adolescence, however, can occur at any age [2]. Family history of autoimmune disease such as coeliac’s disease or Hashimoto’s thyroiditis may lead to higher risk [3]. Genetic predisposition however plays a significant role. Particularly HLA class II alleles, such as HLA-DR3-DQ2 and HLA-DR4-DQ8, increase susceptibility to autoimmune β-cell destruction [4].

Type II diabetes mellitus (T2DM) is commonly seen in individuals over 40 years of age, although increasing prevalence of obesity is leading to increasing diabetes in younger population [5]. Individuals with central obesity, elevated triglycerides, reduced HDL levels and hypertension are particularly at a higher risk [6]. In terms of pathophysiology, T2DM is multi-faceted including insulin resistance, which is often driven by obesity, metabolic syndrome with a role of genetic predisposition [7]. Whilst autoimmune β-cell destruction defines Type I diabetes, β-cell failure and chronic insulin resistance take precedence in T2DM (Fig. 1) [8]. Insulin resistance is exacerbated when excess adipose tissue secretes adipokines and pro-inflammatory cytokines such as TNF- α and IL-6, which interfere with insulin signalling [9]. Thus, reducing glucose uptake by cells and contribute to chronic low-grade inflammation [10]. This insulin resistance causes chronic overproduction of insulin which combined with glucotoxicity, lipotoxicity and amyloid polypeptide (IAPP) accumulation leads to progressive β-cell dysfunction and apoptosis ultimately reducing insulin secretion [11].

**Fig. 1:**
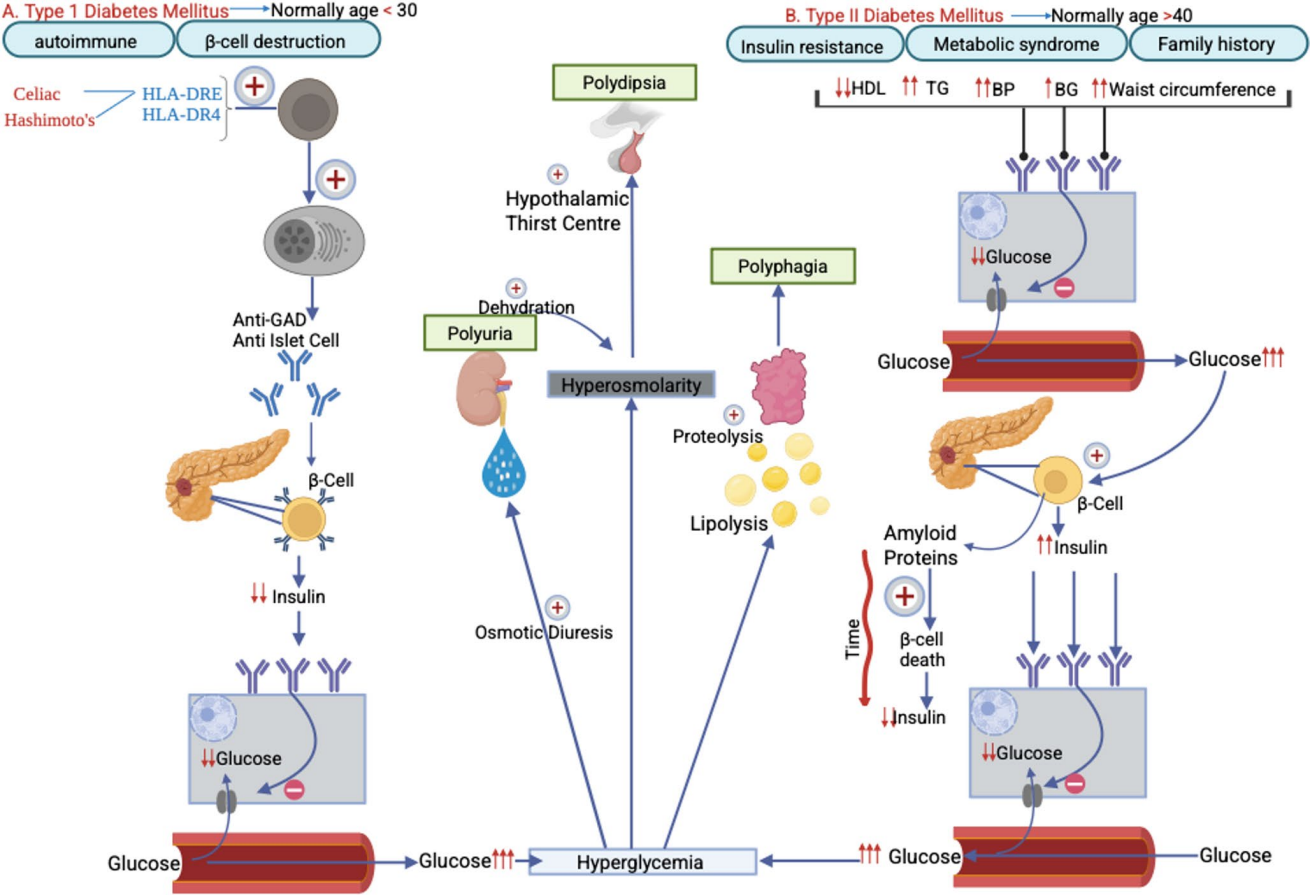
Comparative pathophysiology of Type I and II diabetes: Panel (**A**) shows autoimmune process, often triggered in genetically predisposed individuals (HLA-DR3/DR4), leads to the destruction of pancreatic β-cells by anti-GAD and anti-islet cell antibodies. Which leads to absolute deficiency and prevents glucose uptake into cells. Panel (**B**) shows characterisation by insulin resistance typically occurring after 40 years of age associated with metabolic syndrome and obesity. Initially, β-cells increase insulin output to compensate but with time amyloid accumulation and chronic metabolic stress causes β-cell dysfunction and reduced insulin secretion. In both scenarios, consequent hyperglycaemia causes osmotic diuresis, polyuria, dehydration and compensatory polydipsia. Cell starvation also stimulates lipolysis and proteolysis, resulting in polyphagia. Key terms: GAD- Glutamic Acid Decarboxylase; TG- Triglycerides; HDL- High Density Lipoprotein; BG- Blood Glucose

Obesity represents a worldwide health problem and is associated with many diseases including diabetes mellitus [12]. Obesity is a result of excessive caloric intake in comparison to caloric expenditure. This leads to increased lipogenesis, expansion of adipose tissue depots, particularly visceral fat and chronic metabolic dysregulation [13]. This is all part of the sedentary lifestyle with limited physical activity and dietary habits which include high calorie and high GI index food intake feeding into the dopamine pathway, plays a major role in obesity [14]. Another major contributor to obesity is psychological status including stress and depression [15], which impacts eating behaviour of the patients [16]. This is worsened by medications such as serotonin reuptake inhibitors (SSRIs) and antipsychotics which can increase appetite and alter metabolism thus causing weight gain [17]. Metabolic dysfunctions such as hypothyroidism and Cushing’s syndrome and medication such as corticosteroids can contribute to this imbalance of caloric intake and expenditure however less common [18].

Obesity, central adiposity in particular, is an independent predictor for metabolic syndrome, shaped by convergence of lifestyle factors, metabolic dysregulation and genetic predisposition [19]. A dysregulation in adipokine secretion characterised by elevated pro-inflammatory adipokines (TNF- α, RESISTIN, IL-6) alongside reduced adiponectin compromises insulin signaling and subsequently contributes to hyperglycemia and insulin resistance [20]. Adipokines in conjunction with insulin resistance, activate the renin-angiotensin-aldosterone system (RAAS) and the sympathetic nervous system (SNS) culminating into hypertension, vasoconstriction and sodium retention [21]. Insulin resistance and adipokines drive dyslipidemia by increasing hepatic VLDL production and suppressing HDL levels, which in turn aggravates triglyceride accumulation especially around abdomen [16].

The pancreas, a retroperitoneal organ behind the stomach with both exocrine and endocrine functions is a crucial player in this metabolic regulation [22]. It responds to the above stated systemic disturbances by modulating hormone secretions. The exocrine function of pancreas is producing pancreatic juice using acinar cells which comprise 99% of the pancreatic volume [23]. This juice is rich in enzymes like amylase, lipase and bicarbonates that help digestion of carbohydrates, proteins and fats. The islets of Langerhans constitute the remaining 1% volume of pancreas and they regulate the blood glucose homeostasis as part of pancreas endocrine function [24]. Islets of Langerhans include Alpha, beta, delta and F cells and each cell type plays an individual role in blood glucose regulation [25].

Alpha cells produce glucagon which is a hormone that raises blood glucose levels by stimulating the liver to produce glucose through gluconeogenesis and glycogenolysis and by breaking down fat in adipose tissue [26]. On the other hand, insulin is produced by β-cells [27]. Insulin a well-recognised hormone that lowers blood glucose levels by promoting uptake of glucose into muscles and adipose tissues through the GLUT-4 transporter and by stimulating the production of glucose, glycogen and lipogenesis [28]. Insulin secretion is triggered by increasing glucose levels in blood [29]. Glucose oxidation is directly related to increased ATP production which closes potassium channels leading to cell depolarisation, calcium influx and vesicle fusion to release insulin, c-peptide and amylin [30]. It is the excess of amylin in T2DM that leads to buildup of amyloid deposits and damage β-cells [31]. Delta cells release somatostatin, a hormone that regulates the release of insulin and glucagon [32]. Lastly F cells produce pancreatic polypeptide hormone which partakes in regulating insulin secretion, promoting growth of pancreatic cells and inhibiting release of glucagon [33]. Insulin and glucagon function as opposing regulators of blood glucose homeostasis. Insulin also synergises with other hormones such as epinephrine and norepinephrine. On the other hand, it supports critical processes such as brain development by interacting with thyroid hormone [34]. Thus, pancreas plays a central role in balancing energy production, storage and utilisation and disruption in these regulatory systems can lead to metabolic imbalances, excessive fat storage and weight gain [35].

## Symptoms and complications of diabetes mellitus

Metabolic syndrome often combined with obesity significantly increases the risk of several systemic complications mainly but not limited to cardiovascular, respiratory and neurological functions. Hypertension is one of the most occurring complications which is commonly seen in individuals with obesity and metabolic syndrome [16]. According to American College of Cardiology (ACC) and the American Heart Association (AHA) (2017 guidelines), hypertension is defined as a systolic blood pressure ≥ 130 mmHg or a diastolic blood pressure ≥ 80 mmHg, confirmed on at least two separate occasions [36]. Adipokine dysregulation, particularly elevated leptin, TNF- α and IL-6 stimulate the RAAS and SNS resulting in sodium retention, vasoconstriction, increased cardiac output eventually elevating blood pressure. Hypertensive patients are often asymptomatic and only diagnosed through routine or opportunistic testing [36].

T2DM however presents itself with classic symptoms of hyperglycaemia including polyuria, polydipsia and unexplained weight loss, sometimes accompanied by polyphagia and blurred vision. These symptoms stem from osmotic diuresis and cellular glucose deprivation [37]. A detailed overview of the diagnostic tests, complications, and management strategies for diabetes is presented in Table 1, while the metabolic complications associated with obesity and metabolic syndrome are outlined in Table 2.

**Table 1:** Comprehensive overview of diabetes

Test	Diagnostic Criteria	Condition	Management Strategy	Description
Fasting Plasma Glucose (FPG)	≥ 126 mg/dL on two separate occasions	Diabetes Mellitus	Insulin Therapy for Type I; Metformin as first-line for Type II	GLP-1 agonists, SGLT2 inhibitors, and DPP-4 inhibitors for Type II, especially with ASCVD or CKD(138)
Oral Glucose Tolerance Test (OGTT)	Glucose level ≥ 200 mg/dL two hours after a 75-gram glucose load	Diabetes Mellitus	Lifestyle modifications, oral hypoglycemics, insulin therapy if necessary	Used to diagnose gestational diabetes or impaired glucose tolerance(139)
HbA1c	≥ 6.5% indicates diabetes	Diabetes Mellitus	Glycemic control with oral agents, insulin in severe cases	Lowering HbA1c reduces the risk of complications(140)
Random Blood Glucose	≥ 200 mg/dL with symptoms	Diabetes Mellitus	Immediate glucose control in symptomatic patients	Used for rapid assessment of hyperglycemia(141)
Autoantibodies & C-peptide Levels	Positive autoantibodies, low C-peptide in Type I	Type I vs. Type II Differentiation	Type I: Insulin therapy; Type II: Lifestyle changes, oral agents	Confirms autoimmune diabetes vs. insulin resistance(7)
Diabetic Ketoacidosis (DKA) Markers	pH < 7.3, serum ketones, glucose > 250 mg/dL	Acute Metabolic Crisis	IV fluids, insulin infusions, electrolyte management	Common in Type I diabetes, life-threatening if untreated(142)
Hyperglycemic Hyperosmolar Syndrome (HHS) Markers	Glucose > 600 mg/dL, osmolality > 320 mOsm/kg	Acute Metabolic Crisis	IV fluids, gradual glucose correction	More common in Type II diabetes(39)
Microalbuminuria (Urinary ACR Test)	Albumin-to-creatinine ratio (ACR) ≥ 30 mg/g	Diabetic Nephropathy	ACE inhibitors or ARBs	Helps detect kidney damage early(143)
Fundoscopy & Retinal Imaging	Microaneurysms, hemorrhages, neovascularization	Diabetic Retinopathy	Laser therapy, VEGF inhibitors	Prevents vision loss in diabetic patients(144)
Peripheral Nerve Testing	Loss of sensation, vibratory sense	Diabetic Neuropathy	Pain management, foot care	Prevents foot ulcers and infections(145)
Lipid Panel	Triglycerides > 150 mg/dL, low HDL, high LDL	Atherosclerosis Risk in Diabetes	Statins, lifestyle changes	Essential in managing cardiovascular risks(146)

**Table 2:** Comprehensive overview of obesity and metabolic syndrome [147]

Test	Diagnostic Criteria	Condition	Management Strategy	Description
Body Mass Index (BMI)	BMI ≥ 30 kg/m	Obesity	Lifestyle modifications, pharmacotherapy, bariatric surgery	BMI categories: Class 1 (30–35), Class 2 (35–40), Class 3 (> 40)
Waist Circumference	> 40 inches (males), > 35 inches (females)	Abdominal Obesity	Weight loss, exercise	Central obesity is a key marker of metabolic syndrome
Blood Pressure Measurement	≥ 130/85 mmHg	Hypertension in Obesity	ACE inhibitors, ARBs, diuretics	Obesity-induced hypertension worsens cardiovascular risks
Lipid Panel	Triglycerides > 150 mg/dL, HDL < 40 mg/dL (males), < 50 mg/dL (females)	Dyslipidemia in Obesity	Statins, fibrates	Obesity leads to high LDL and low HDL, accelerating ASCVD
Fasting Plasma Glucose (FPG)	100–125 mg/dL (prediabetes)	Metabolic Syndrome	Diet, exercise, Metformin for high-risk individuals	Prediabetes is an early warning for Type II diabetes
Liver Function Tests (LFTs)	Elevated AST/ALT, AST/ALT ratio < 1	Nonalcoholic Fatty Liver Disease (NAFLD)	Weight loss, GLP-1 agonists, Pioglitazone	Excess fat accumulation in hepatocytes leads to liver fibrosis
Polysomnography (Sleep Study)	Apnea-hypopnea index (AHI) > 5 events per hour	Obstructive Sleep Apnea (OSA)	CPAP therapy, weight loss, surgery in severe cases	Fat deposition around the neck leads to airway obstruction and nocturnal hypoxia
Arterial Blood Gas (ABG) Analysis	pCO > 45 mmHg with hypoxia	Obesity Hypoventilation Syndrome (OHS)	Non-invasive ventilation, weight loss, respiratory therapy	Severe obesity impairs breathing and gas exchange
Cardiac Stress Test	Exercise-induced ischemia	Cardiovascular Disease in Obesity	Lifestyle changes, β blockers, statins	Detects early signs of CAD linked to obesity

Acute complications of diabetes arise suddenly and require immediate medical intervention. One of the most life-threatening conditions is Diabetic Ketoacidosis (DKA), which predominantly affects individuals with type I diabetes. A triggering stressor, such as infection or insulin non-compliance, leads to an increase in counter-regulatory hormones (glucagon, cortisol, catecholamines, growth hormone), driving excessive hepatic glucose production through gluconeogenesis and glycogenolysis, resulting in severe hyperglycaemia. The lack of insulin results in excessive activation of hormone-sensitive lipase, promoting unregulated lipolysis. Free fatty acids are converted into ketone bodies (β-hydroxybutyrate and acetoacetate) in the liver, leading to metabolic acidosis. Patients present with Kussmaul breathing, dehydration, fruity-smelling breath and electrolyte imbalances, particularly potassium shifts. Although serum potassium may initially appear normal or elevated due to acidosis, total body potassium is typically depleted. If untreated, DKA can progress to coma and eventually death [38].

Another acute metabolic crisis is Hyperglycemic Hyperosmolar Syndrome (HHS), which is more common in T2DM. Characterized by extreme hyperglycemia and profound dehydration due to osmotic diuresis, HHS differs from DKA in that residual insulin activity suppresses lipolysis and ketogenesis, preventing significant ketone accumulation. Symptoms include confusion, severe dehydration and altered mental status, which can progress to coma if not managed promptly [39]. Diabetic CN III palsy is an acute neurological complication caused by ischemic damage to the vasa nervorum, affecting the somatic fibers of cranial nerve III while sparing the parasympathetic fibers. This results in characteristic “down and out” eye position with pupil sparing. These acute metabolic and vascular complications are further categorized in Table 1, where their diagnostic markers and treatment approaches are systematically presented [40].

In contrast, chronic complications of diabetes develop gradually due to prolonged hyperglycaemia, affecting multiple organ systems. One of the most serious is Atherosclerotic Cardiovascular Disease (ASCVD), which result from nonenzymatic glycation of LDL molecules, increased triglycerides and decreased HDL levels, accelerating atherosclerosis. This increases the risk of transient ischemic attacks (TIAs), coronary artery disease (CAD), strokes, myocardial infractions (MIs) and peripheral artery disease (PAD) [41]. The vascular damage extends to the kidneys leading to Diabetic Neuropathy, where persistent hyperglycaemia damages the glomerular filtration system, causing proteinuria, reduced glomerular filtration rate (GFR) and ultimately chronic kidney disease (CKD) [42]. Albuminuria serves as an early indicator of renal dysfunction. Similarly, Diabetic neuropathy arises from microvascular damage in the retina, progressing from microaneurysms and haemorrhages to neovascularization, which can cause vitreous haemorrhage and vision loss.

Diabetic Neuropathy is another progressive complication caused by osmotic damage to Schwann cells and neurons, leading to both sensory and autonomic nerve dysfunction [43]. Symptoms include numbness and tingling in a stocking-glove distribution, gastroparesis, orthostatic hypotension and foot ulcers that are highly susceptible to infection. A structured comparison between diabetes related complications and obesity related metabolic disturbances can be seen in Table 2, highlighting their distinct yet overlapping consequences [42].

Beyond metabolic and vascular complications, obesity contributions to respiratory and hepatic disorders. Obstructive Sleep Apnea (OSA) and Obesity Hypoventilation Syndrome (OHS) are direct consequences of excessive adipose deposition around the neck and thorax, which leads to airway compression, nocturnal hypoxia, and chronic hypercapnia (pCO_2_ > 45 mmHg). These conditions significantly impair sleep quality and oxygenation, increasing the risk of cardiovascular and metabolic disturbances [44].

Another major consequence of obesity- related insulin resistance is Non-alcoholic Fatty Liver Disease (NAFLD), where excessive hepatic lipogenesis leads to fat accumulation within hepatocytes (steatosis). Overtime, this can progress to non-alcoholic steatohepatitis (NASH) and hepatic fibrosis, culminating in cirrhosis. NAFLD is often detected through mildly elevated liver enzymes (AST/ALT ratio < 1) and increased liver echogenicity on ultrasound [45]. These metabolic complications, which are distinct from but often associated with diabetes, are summarized in Table 2, emphasising the role of obesity in driving systemic disease.

Given the wide spectrum of complications associated with obesity, metabolic syndrome and diabetes, early diagnosis and comprehensive management are essential. Acute complications like DKA and HHS require immediate medical intervention, while chronic conditions such as ASCVD, nephropathy and neuropathy necessitate long term monitoring and therapeutic strategies to prevent irreversible damage. The interplay between metabolic dysregulation, vascular disease and organ dysfunction underscores the importance of lifestyle modifications, pharmacologic interventions and routine medical surveillance in reducing morbidity and mortality.

Tables 1 and 2 outline the differences in diagnostic approaches, complications and management strategies between diabetes and obesity related metabolic disorders [37].

Diabetes mellitus is a complex health condition with long-lasting and significant implications for health. Early recognition, understanding pathophysiology and appropriate treatment, and lastly avoiding complications can improve outcomes. It needs a holistic approach including lifestyle changes, appropriate and timely pharmacotherapy and continuous and regular monitoring are integral in managing it [46].

## Epigenetics of type 2 diabetes mellitus

Epigenetics plays a crucial role between the genetic and environmental factors influencing type 2 diabetes mellitus. Epigenetic modifications are mediated by DNA methylation, histone modifications and noncoding RNA’s, which regulate key biological processes including insulin secretion, resistance and inflammatory changes all of which contribute to pathogenesis of type 2 diabetes mellitus. These are heritable changes in gene expression that do not alter DNA sequence. Among others oxidative stress plays a central role in modifying the epigenome. Reactive oxygen species (ROS) production is increased by hyperglycaemia and lipotoxicity, which disrupts the above-mentioned DNA methylation, histone acetylation and noncoding RNA activity thereby altering β-cell function and inflammatory responses. Therapeutic opportunities arise by understanding these mechanisms such as interventions like polyphenols and metformin which have antioxidant and epigenetic-modulating properties [47].

## Epigenetic mechanisms in T2DM pathogenesis

Epigenetics modifications play a central role in β-cell function, insulin resistance, and systemic inflammation of T2DM. As shown in Fig. 3, metabolic stress initiates a self-amplifying cascade of epigenetic modifications i.e., DNA methylation, histone acetylation and miRNA dysregulation that gradually compromises β-cell identity and insulin action.

## DNA methylation and gene regulation

**Fig. 2:**
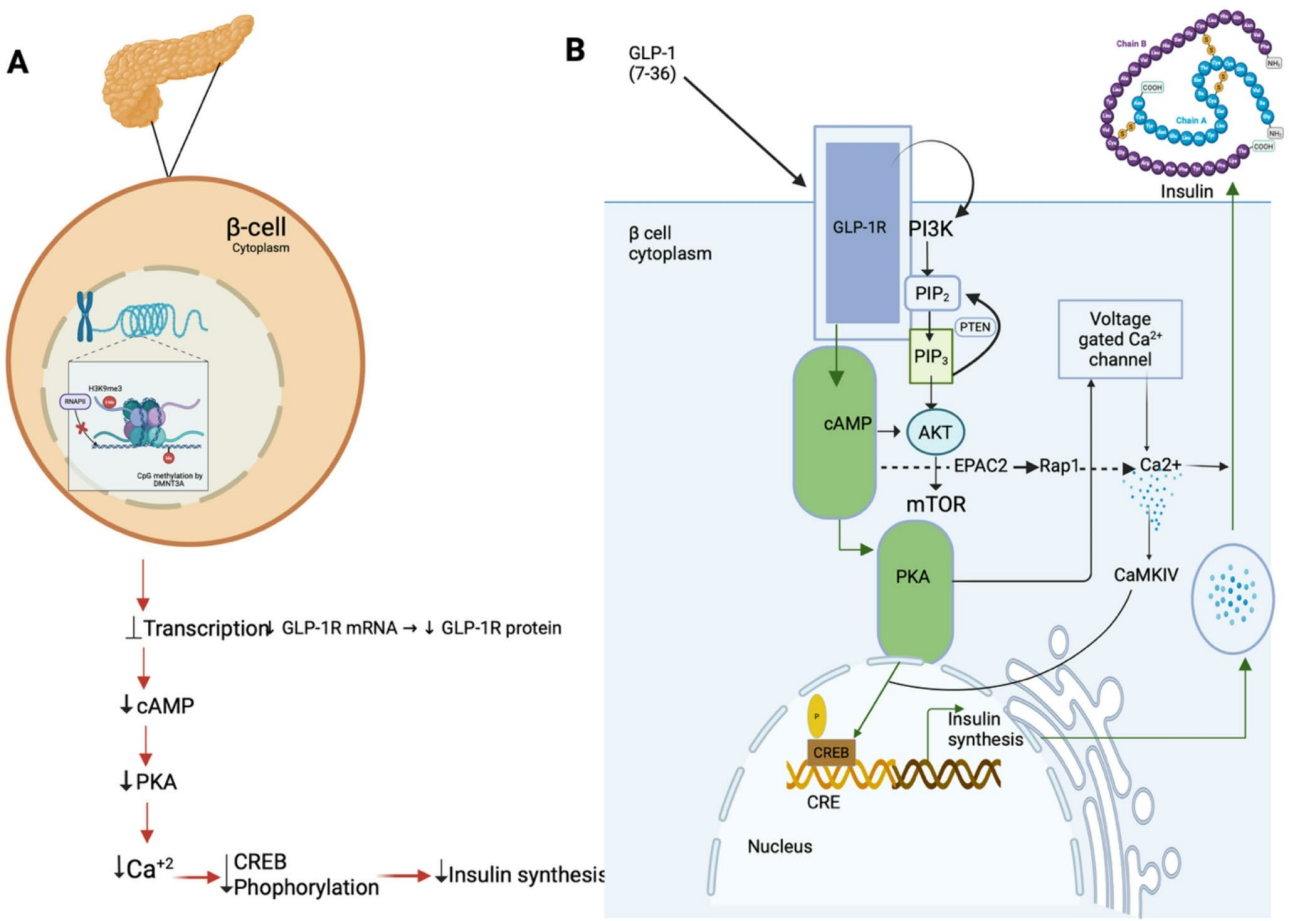
Epigenetic silencing of GLP-1R impairs incretin signaling and insulin secretion in pancreatic β-cells. **A** Under diabetogenic or metabolic-stress conditions, recruitment of DNMT3A to the GLP1R promoter deposits repressive CpG methylation (Me) and reduces H3K4me3, leading to diminished GLP-1R mRNA and protein levels. Loss of receptor expression blunts GLP-1 [7–36]-stimulated cAMP generation, attenuates PKA activation, decreases voltage-gated Ca²⁺ influx and CaMKIV signaling, and reduces CREB phosphorylation at the CRE in the nucleus—collectively suppressing insulin gene transcription and peptide synthesis. **B** In healthy β-cells, GLP-1 binding to GLP-1R engages Gₛ to activate adenylyl cyclase and raise intracellular cAMP. cAMP then triggers two complementary pathways: [1] PKA-mediated phosphorylation of CREB to drive insulin gene transcription at the CRE site; and [2] EPAC2–Rap1–mTOR signalling (dashed arrow) alongside PI3K-dependent conversion of PIP₂ to PIP₃ (negatively regulated by PTEN) and downstream AKT activation. Concurrently, PKA enhances voltage-gated Ca²⁺ channel opening, while CaMKIV amplifies the Ca²⁺-driven exocytotic machinery. The combined effects of PKA, EPAC2 and PI3K/AKT pathways ensure robust CREB activation, insulin synthesis, and granule release Abbreviations: Abbreviations: GLP-1R, glucagon-like peptide-1 receptor; DNMT3A, DNA methyltransferase 3 A; PKA, protein kinase A; CaMKIV, Ca²⁺/calmodulin-dependent kinase IV; CREB, cAMP response element–binding protein; CRE, cAMP response element; PI3K, phosphoinositide 3-kinase; PIP₂, phosphatidylinositol-4,5-bisphosphate; PIP₃, phosphatidylinositol-3,4,5-trisphosphate; PTEN, phosphatase and tensin homolog; AKT, protein kinase B; EPAC2, exchange protein directly activated by cAMP 2;mTOR, mechanistic target of rapamyci

DNA methylation is a master regulator mechanism leading to gene silencing. It drives gene transcription by the addition of methyl groups to cytosine-phosphate-guanine (CPG) islands. This process operates through DNA methyltransferases (DNMTs) such as DNMT1, DNMT3B and DNMT3A, which are critical to maintain epigenetic viability [48]. Aberrant DNA methylation patterns have been linked to β-cell dysfunction and attenuated glucose metabolism of T2DM. A salient example is hypermethylation of PDX1 gene which is integral to β-cell differentiation and transcriptional regulation of insulin [49]. This modification represses PDX1 expression and contributes to compromised insulin secretion and lowers β-cell survival rate [50]. Comparably, PAX4 gene shows altered methylation in T2DM which is normally essential for maintaining β-cell lineage restriction. This alteration mitigates β-cell stability and elevates susceptibility to dedifferentiation further compromising insulin production. DNMT3A induced hypermethylation of glucagon-like peptide-1 receptor (GLP-1R) promoter silences receptor expression thereby reduces GLP-1 driven cAMP/PKA signalling and hence insulin secretion (Fig. 2) [51]. In addition to its established role in telomere dynamics and glucose metabolism, epigenetic regulation has been identified as significant contributors to the aetiology of diabetic kidney disease (DKD) and end-stage kidney disease (ESKD) observed predominantly in individuals with type 1 diabetes (T1D). Telomere shortening is a well-established marker of faster biological aging and has been linked to DKD process [52]. Although many of these findings originate from T1D studies, similar metabolic stressors in T2DM are known to induce comparable epigenetic alterations. These stressors include chronic hyperglycaemia, oxidative stress and inflammation. Epigenetic modifications notably anomalous DNA methylation in telomerase-associated genes in particular MAD1L1 and TUBB further highlight the involvement of epigenetic dysregulation in renal dysfunction [53]. These genes, indispensable to cellular pathways like Wnt signalling, are often disrupted in DKD [54]. Mechanistic link between epigenetic modifications and progressive renal dysfunction in diabetes is highlighted by the above findings.

## Histone modifications and β-cell dysfunction

**Fig. 3:**
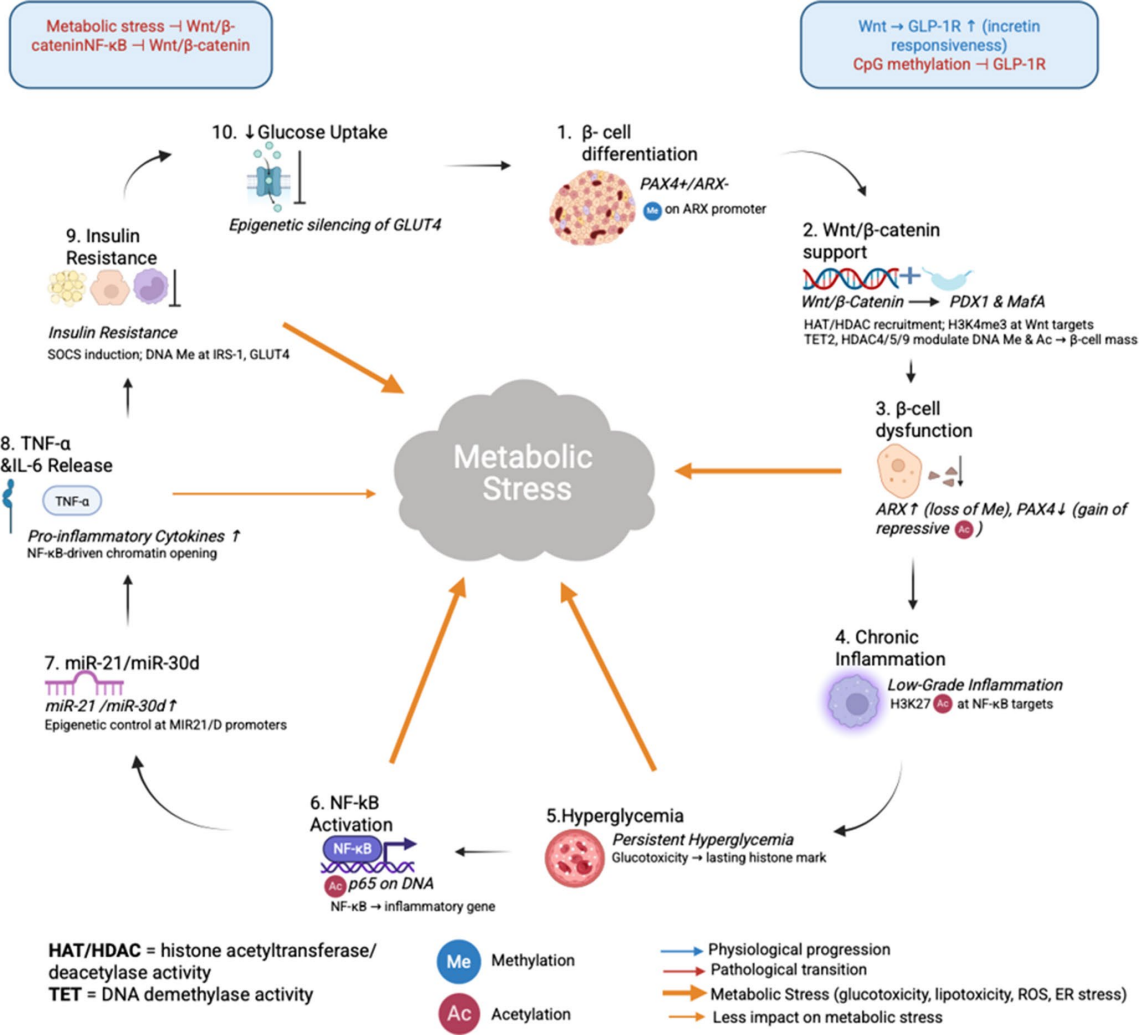
Epigenetic circuitry driving metabolic-stress-induced β-cell failure and insulin resistance in diabetes. Under physiological conditions (blue arrows), Wnt/β-catenin signaling promotes β-cell differentiation [1] via PDX1/MafA activation and coordinated histone acetyltransferase (HAT) recruitment and TET-mediated DNA demethylation, while PAX4/ARX balance is maintained by promoter methylation. Metabolic stressors—including glucotoxicity, lipotoxicity, ROS and ER stress (thick orange arrows)—disrupt Wnt/β-catenin and GLP-1R signaling (boxed insets), triggering a cascade of epigenetic alterations: 1. Loss of Wnt support leads to ARX upregulation (loss of Me) and PAX4 silencing (gain of repressive Ac), driving β-cell dysfunction [3]. 2. Chronic low-grade inflammation [4] is maintained by H3K27 acetylation at NF-κB target loci, amplifying TNF-α/IL-6 releases [8] through NF-κB–driven chromatin opening. 3.Persistent hyperglycemia [5] leaves lasting histone marks that further prime inflammatory gene transcription via p65 acetylation [6] and upregulation of miR-21/miR-30d [7] at their promoters. 4.Insulin resistance [9] ensues from SOCS induction and DNA methylation at IRS-1 and GLUT4, culminating in epigenetic silencing of GLUT4 and reduced glucose uptake [10]. Together, these interconnected epigenetic events establish a self-reinforcing pathological loop that undermines β-cell identity, perpetuates inflammation and insulin resistance, and accelerates diabetes progression. Me, DNA methylation; Ac, histone acetylation; HAT/HDAC, histone acetyltransferase/deacetylase; TET, DNA demethylase

Histone modifications alter chromatin structure in a manner that either induces or silences gene expression [55]. In T2DM, inflammatory responses and insulin secretion are substantially influenced by histone acetylation and deacetylation. Histone acetyltransferases (HATs) stimulate acetylation which boosts transcription of core genes such as insulin gene (INS) which thereby facilitates insulin synthesis [56]. Reciprocally, histone deacetylases (HDACs) promote transcriptional repression by removing acetyl groups. HDACs are hyperactive in T2DM causing β-cell dysfunction and reduced insulin secretion. Evidence implies that HDAC inhibitors can counteract this effect thus positioning them as emerging therapeutic agents [57]. Histone modifications further influence inflammation markedly via their effect on adipokine regulation. These changes include Neureglin 4 [58] and omentin [59] both of which are fundamental for supporting insulin sensitivity and securing overall metabolic health. Dysregulation of histone modifications exacerbate insulin resistance by altering adipokine expression and promoting chronic inflammation. The dynamic interaction between histone modifications, metabolic dysfunction and inflammation draws attention to the importance of epigenetic regulation in T2DM progression [60] (Fig. 3).

## Noncoding RNAs and insulin resistance

Noncoding RNAs, particularly microRNA (miRNAs) and long noncoding RNAs (lncRNAs), are master regulators of inflammation, β-cell survival and insulin sensitivity within T2DM. These regulatory RNAs modulate gene expression at the post-transcriptional level by interacting with target mRNAs, leading to either their degradation or translational inhibition [61]. For instance, substantial evidence implicates both miR-21 and miR-146a in the pathophysiological processes of diabetes [62, 63]. Under diabetic conditions, miR-21 is upregulated in pancreatic β-cells, promoting β-cell apoptosis through targeting of anti-apoptotic genes like PDCD4 and BCL2, which results in decreased β-cell mass and insulin secretion [28]. This process drives β-cell failure, which is recognised as a hallmark of disease progression. Likewise, miR-146a exerts a dual function in both insulin resistance and inflammation [64]. Through the alteration of mitochondrial function and disruption of inflammatory pathways, miR-146 A compromises insulin signalling across adipose tissue, skeletal muscle and liver worsening glucose tolerance [28].

Alongside miRNAs, lncRNA have recently emerged as important modulators of T2DM. Recognised as a well-studied lncRNA, MALAT1 exerts substantial effects on inflammation and insulin resistance [65]. Under diabetic conditions, MALAT1 shows elevated expression in adipose tissues and skeletal muscle. It triggers the NF-κB signalling pathway, a central regulator of inflammatory responses. MALAT1 enhances NF-κB mediated cytokine release, contributing to sustained low grade inflammation thereby aggravating insulin resistance within insulin responsive tissues [28]. These findings imply that lncRNAs and miRNAs contribute to metabolic disturbances in T2DM and could be targeted therapeutically to reestablish insulin sensitivity and support β-cell survival [66].

## Epigenetics of β-Cell dysfunction and insulin resistance

Epigenetic changes are central to the progression of T2DM, as it drives two hallmark features of the disease, β-cell dysfunction and insulin resistance. These alterations affect the expression of essential genes controlling β-cell identity, inflammatory pathways and insulin secretion which in turn hastens disease progression and exacerbates metabolic dysregulation [30, 31].

### β-Cell Dysfunction and dedifferentiation

The identity and function of β-cells are precisely controlled through the coordinated expression of ARX and PAX4 genes [67]. PAX4 plays a vital role in both the development and sustained function of pancreatic β-cell, ensuring their capacity to generate and secrete insulin. In contrast, ARX is a transcription factor essential for alpha-cell lineage specification, with its expression typically suppressed in mature β-cell [68].However, during T2DM, epigenetic regulation disrupts this balance, inducing the phenomenon of β-cell dedifferentiation, wherein β-cell lose their specialised insulin secreting identity and adopt alpha like cells features. This phenomenon is primarily driven by elevated ARX expression, redirecting β-cell towards a glucagon producing phenotype, which in turn diminishes insulin secretion and disrupts glucose homeostasis [69]. At the same time, decreased PAX4 expression exacerbates the loss of β-cell identity, impairing their insulin production and capacity to control blood glucose levels [30].

Oxidative stress, a key mediator of epigenetic alterations, amplifies these changes. Chronic hyperglycaemia and lipotoxicity drive overproduction of reactive oxygen species (ROS), thereby disturbing the epigenetic landscape of β-cells through modifications of DNA methylation and histone modifications [70]. These modifications driven by oxidative stress heighten β-cells vulnerability to glucotoxicity and lipotoxicity, contributing to a more rapid decline in their function [30]. Progressive loss of β-cell identity, together with oxidative stress and epigenetic alterations, substantially contribute to pancreatic β-cell failure, a defining trait of T2DM.

Insulin Resistance and Systemic Inflammation Systemic low-grade inflammation significantly contributes to insulin resistance, and its levels are tightly controlled by epigenetic modifications. In normal physiological states, insulin engages its receptor, initiating a downstream signalling cascade that promotes glucose uptake into insulin sensitive tissues including liver, skeletal muscle and adipose tissue [71]. However, in T2DM patients, persistent inflammation interferes with the process, resulting in dysfunctional insulin signalling and decreased glucose utilisation [31]. A key pathway implicated in inflammation induced insulin resistance is the nuclear factor-kappa B (NF-κB) signaling pathway. Elevated glucose levels, frequently seen in diabetes, trigger aberrant DNA methylation and histone acetylation in the NF-κB p65 promoter, which in turn results in NF-κB activation [72]. This persistent activation leads to increased expression of pro-inflammatory target genes, including tumor necrosis factor alpha (TNF-α) and interleukin-6 (IL-6) [31]. These cytokines drive inflammation and concurrently disrupt insulin receptor signalling, leading to impaired insulin sensitivity in adipose tissue, as well as liver and skeletal muscle. Consequently, glucose uptake in peripheral tissue becomes impaired, resulting in sustained hyperglycaemia and insulin resistance [73].

Apart from NF-κB signaling, miRNAs are essential for mediating inflammation initiated by insulin resistance. miR-30d and miR-21 have both been recognized as critical regulators of this process. miR-30dregulates insulin sensitivity and inflammation through modulation of gene expression which plays a role in immune signaling [74]. Concurrently, miR-21 expression is increased under diabetic conditions, promoting chronic inflammation and insulin resistance by stimulating pro-inflammatory cytokines production [75]. Such persistent inflammatory environment further disrupts insulin action and aggravates metabolic dysfunction, fueling a self-reinforcing cycle of insulin resistance and hyperglycemia.

## Transgenerational epigenetics inheritance in T2DM

A growing body of evidence highlights how epigenetic factors contribute to onset and transgenerational transmission of T2DM. Studies exploring the effects of maternal and intrauterine nutrition indicate a significant association between early developmental environmental conditions and diabetes risk in different species [76]. For instance, investigations into the Dutch Hunger Winter famine demonstrated that those exposed to famine prenatally during the Dutch Hunger Winter of 1944-45 period displayed significantly reduced DNA methylation at the imprinted insulin-like growth factor 2 (IGF2) gene locus six. Decades afterwards, compared to unexposed siblings of the same sex. This association was unique to periconceptional exposure, highlighting the critical importance of early mammalian developmental window in the establishment and maintenance of epigenetic marks [77]. This phenomenon described as “thrifty phenotype hypothesis” indicates that nutritional deprivation during sensitive developmental periods induces long lasting changes in glucose homeostasis. Similarly, maternal overnutrition including gestational diabetes and a high fat diet have been found to impair offspring metabolic outcomes. These parental metabolic perturbations correlate with specific epigenetic marks in progeny, suggesting an epigenetic basis underlying the transgenerational metabolic disease risk [78].

Barker et al.’s landmark (1993) landmark work first articulated the connection between early metabolic risk and prenatal life, thereby inspiring the formulation of the **“Foetal Origins of Adult Disease” (FOAD)** hypothesis. Per this framework, early life exposures such as growth restriction and intrauterine malnutrition, which cause low birth weight, predispose individuals to cardiovascular and metabolic diseases associated with T2DM and insulin resistance [79]. Support for this hypothesis comes from research in both humans and animals, and indicate that hostile intrauterine conditions trigger epigenetic changes within foetal tissues and drive chronic metabolic dysfunction [80].

Evidence from animal models suggest that epigenetic modifications including DNA methylation and histone changes, can be transmitted to offspring in the absence of initial inducing environmental cues. However, the extent and persistence of such mechanisms in humans remain under active investigation. For instance, the F1 and F2 progeny of rats exposed to a protein-deficient diet manifest endothelial dysfunction and high blood pressure, supporting the model of transgenerational epigenetic inheritance [81]. These results demonstrate that epigenetic alterations in germline cells may contribute to transgenerational metabolic phenotypes. Although distinguishing true transgenerational inheritance from shared environmental effects remain challenging in human studies. Suboptimal nutrition in utero as well as postnatal mental health hinder key metabolic function and organogenesis, making offspring more susceptible to metabolic syndrome and diabetes. Furthermore, environmentally-induced germline epigenetic alterations can be transmitted across generations, establishing a lingering disease cycle [82]. Despite compelling data, the field of transgenerational epigenetic inheritance is still under scrutiny, largely due to its relative infancy. Despite its novelty, the expanding literature demonstrates that early life conditions sculpt metabolic health and that epigenetic changes are increasingly recognised as potential contributor to familial heritable risk of diabetes, although causal mechanisms in human are still being defined [76].

**Fig. 4:**
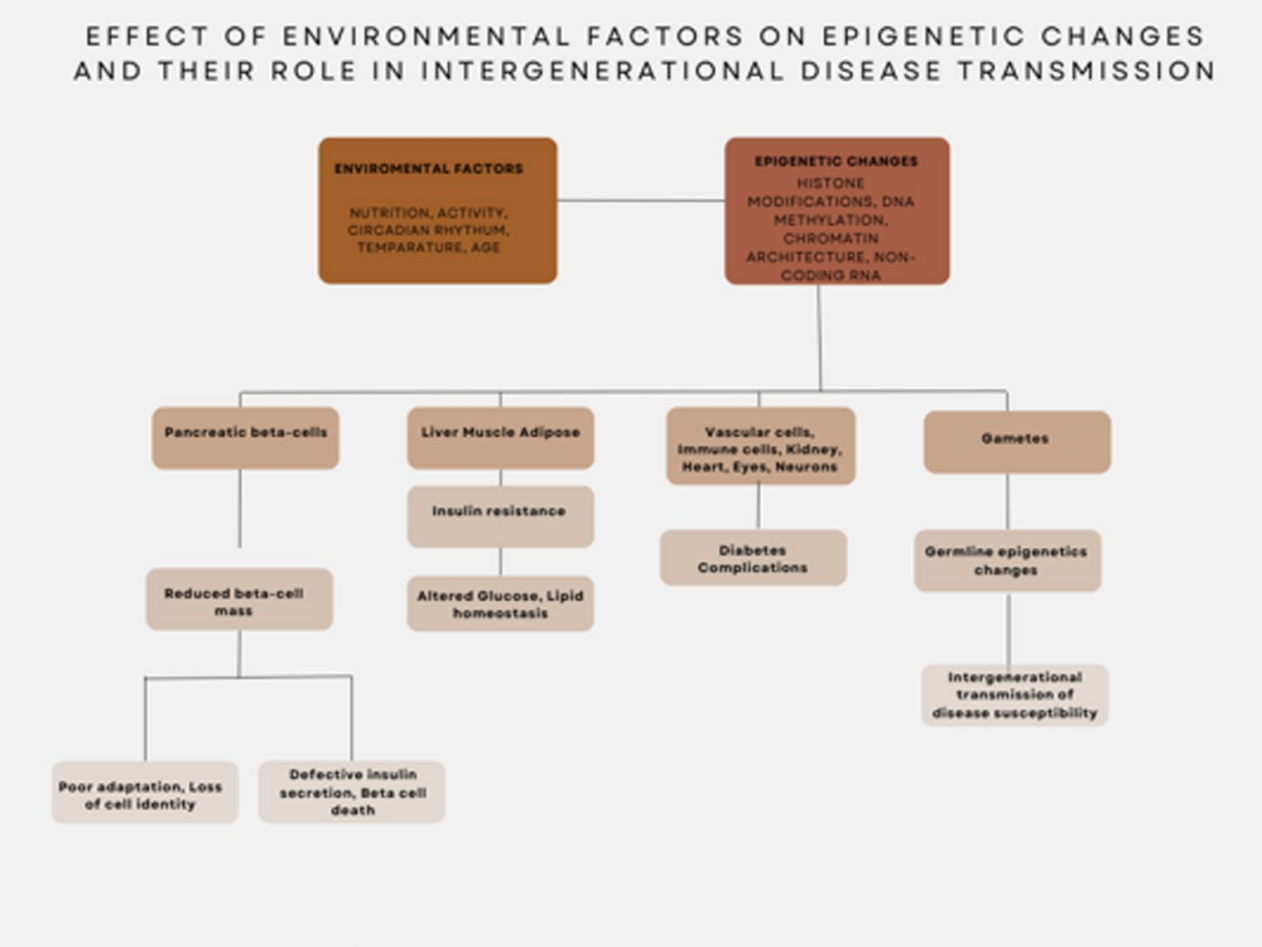
Effect of environmental factors on epigenetic changes and their role in intergenerational disease transmission

While maternal health and its contribution to offspring disease risk are well documented, emerging results underscore the influence of the paternal epigenome. Nutritional imbalance during intrauterine development can alter the epigenetic landscape of male germ cells and impact gene expression programs in the offspring [83]. Findings show that paternal diet modulates lipid and cholesterol in the progeny. In addition, obesity as well as overnutrition and undernutrition in fathers drive reprogramming of the sperm epigenome, and mediate cross-generational modulation of offspring metabolic homeostasis. These data demonstrate that parental metabolic milieu and lifestyle factors elicit epigenetic reprogramming that influences metabolic performance across generations [84]. Moreover, epigenetic perturbations at imprinted genes, which exhibit differential expression from maternal and paternal alleles, play a key role in determining heritable diabetes risk. Not only do epigenetic modifications translate environmental influences into developmental complications of T2DM, but they also contribute to its transgenerational inheritance (Fig. 4). These insights underscore the critical role of epigenetics in the linking environmental exposure, metabolic programming and diabetes risk across generations.

## Clinical implications and epigenetic therapies

The reversibility of epigenetic modifications offers a groundbreaking therapeutic opportunity for handling T2DM. Unlike conventional therapies that primarily focus on glycemic control, epigenetic interventions may alter the trajectory of disease progression at the molecular level, targeting the root causes of insulin resistance, β-cell dysfunction and chronic inflammation [85]. Research into DNA methylation inhibitors, histone modification regulators and micro-RNA based therapies have gained momentum as these strategies aim to restore normal gene expression, improve insulin sensitivity and reduce metabolic inflammation, potentially offering long-term benefits beyond symptom management [41].

## DNA methylation (DNMT) inhibitors

Among the most encouraging interventions for T2DM are DNMT inhibitors, which prevent hypermethylation of key metabolic genes. DNA methylation serves as a fundamental epigenetic regulator of gene expression, either upregulating or silencing critical genes implicated in glucose metabolism, mitochondrial dynamics and β-cell maintenance. In T2DM, altered methylation patterns of key genes contribute to defective β-cell insulin secretion and exacerbate metabolic imbalance. PDX1 hypermethylation is frequently observed in diabetes, silencing this vital β-cell transcription factor contributes to impaired insulin secretion and reduced β-cell mass [41].

In addition, hypermethylation of PGC1α, which governs oxidative metabolism and mitochondrial biogenesis, is observed in tissues exhibiting insulin resistance. Downregulation of PGC1α impairs mitochondrial ATP synthesis, results in reduced β-cell energy and aggravates insulin resistance [86]. Research shows that DNMT inhibitors reverse aberrant methylation, reengaging PDX1 and PGC1α expression, which enhance insulin output, rejuvenate mitochondrial performance and increase β-cell resilience [41]. These results highlight DMNT inhibitors as groundbreaking therapeutic agents that could alter T2DM pathogenesis at its source instead of traditional hyperglycaemia management.

## Histone deacetylase (HDAC) inhibitors

Histone deacetylase (HDAC) inhibitors represent another significant group of epigenetic regulators, acting on chromatin organization and regulation of transcriptional activity. HDACs catalyse the removal of acetyl moieties from histones, fostering tighter chromatin packaging and inhibition of gene expression [87]. Elevated HDAC function in T2DM contributes to insulin resistance, mitochondrial impairment and β-cell dysfunction [88]. HDAC- mediated suppression of insulin gene expression promotes β-cell impairment and metabolic abnormalities [89].

By targeting HDACs, promoter histone acetylation of target metabolic genes increases as a direct result of enhanced histone acetylation and chromatin accessibility. Research demonstrates that HDAC inhibitor foster β-cell regenerative capacity [90], upregulates insulin gene transcription [91] and boost mitochondrial metabolic function [92]. Recognised for its HDAC inhibitor activity, curcumin facilitates improved insulin sensitivity, reduced oxidative stress and suppressed pro-inflammatory cytokines (TNF- α, IL-6) that exacerbate insulin resistance [93]. Preclinical models suggest that curcumin and other HDAC inhibitors could be used to restore β-cell function, enhance glucose metabolism and reduce systemic inflammation, making them a potential therapeutic strategy in T2DM therapy [94].

## microRNA (miRNA)-based therapies

Alongside epigenetic changes to DNA and histones, miRNAs act as powerful post-transcriptional modulator of gene expression, and controllers of β-cell longevity, insulin release and inflammatory responses [95]. MicroRNAs – approximately 22 nucleotides in length – recognise and bind specific mRNA sequences, regulate gene expression by promoting mRNA degradation or inhibiting translation, thereby fine tuning metabolic and immune response in T2DM [96].

Among the many aberrantly expressed miRNAs implicated in diabetes, miR-21 and miR-146a have emerged as key contributors to β-cell dysfunction and metabolic inflammation [97]. To miR-21, which is consistently upregulated in diabetic individuals, induces β-cell apoptosis through suppression of anti-apoptotic genes like PDCD4 and BCL2. This contributes to a progressive β-cell mass depletion, which further impairs insulin secretion and worsens metabolic imbalance [98]. Similarly, miR-146a disrupts mitochondrial function and inflammatory pathways, and aggravates insulin resistance by impairing insulin receptor signalling in insulin-responsive organs such as liver, skeletal muscle and adipose depots [99].

To counteract these detrimental effects, anti-mRNA therapies have emerged as promising strategies for mitigating β-cell apoptosis and enhancing insulin sensitivity. More specifically, targeting miR-21 and miR-146a, which are both unregulated under diabetic conditions and implicated in inflammatory signalling, by antisense-mediated knockdown has demonstrated protective effects on pancreatic β-cell and peripheral insulin response [100]. Conversely, miRNA mimics are employed to restore the function of downregulated, protective miRNAs, thus promoting β-cell regeneration, suppressing chronic inflammation and restoring glucose homeostasis [101]. Notably, miR-7 and miR-375 are integral to pancreatic islet development and insulin secretion, while miR-200 family members, particularly miR-200c, exhibit anti-inflammatory and anti-fibrotic properties that counter metabolic dysregulation [102]. Additional beneficial miRNAs, including miR-30d and miR-124a, promote insulin gene expression and maintain islet architecture [103, 104]. Given their precise post-translational regulatory roles and tissue specificity, miRNA-based therapeutics represent a novel and powerful avenue for reversing the molecular disturbances of T2DM, thereby offering a more effective disease-modifying intervention.

## Ongoing clinical trials and personalised medicine

As interest in epigenetic-based therapies expands, researchers are actively exploring its clinical potential for predicting disease progression and guiding personalised treatment strategies. Efforts are underway in clinical research to harness epigenetic biomarkers as tools for patient stratification, with the goal of enabling highly individualised, biomarker-guided therapy [105]. For example, in a stratified treatment framework, DNMT inhibitors may offer therapeutic advantage for those with hypermethylated PDX1 and PGC1αwhile patients with elevated HDAC activity could experience improved outcome with HDAC inhibitors [100, 102]. Similarly, individuals with dysregulated miRNA expression may be suitable candidates for anti-miRNA or miRNA-mimic therapies, representing precision medicine strategies that transcend conventional one-size-fits-all treatment models [104].

Despite the compelling promise of epigenetic interventions, several key challenges must be addressed before their complete integration into clinical practice. A substantial limitation resides in the precision of epigenetic drugs as broad spectrum inhibitors may yield off target effects, as an unintended consequence affecting the expression of essential genes not implicated in T2DM. In addition, lack of comprehensive data on their long-term use, suboptimal mechanisms of delivery and the need of economically feasible treatment platforms present salient barriers to its widespread clinical adoption. In spite of that, continued discoveries in epigenetics research offer the potential to transform diabetic care. The integration of epigenetic profiling into healthcare decision making could enable the development of precision therapies targeting directly the molecular mechanisms underpinning T2DM, progressing from symptomatic management towards disease modification [41].

## Wnt/β-Catenin signalling and epigenetics in T2DM

The Wnt/β-catenin signalling pathway serves as a central regulator of pancreatic β -cell development, function, and survival, playing a crucial role in insulin secretion and the maintenance of glucose homeostasis [106]. In its on state, the pathway exhibits dynamic regulatory capacity, orchestrating β -cell differentiation, proliferation and metabolic adaptation in response to physiological and environmental stimuli. Growing evidence suggests that epigenetic changes strongly influence the activity of Wnt/ β-catenin pathway thereby showing a mechanistic connection between T2DM pathogenesis and epigenetic mechanisms. These findings shed light on the therapeutic potential of targeting epigenetic mediators in this pathway to conserve β-cells integrity and regain metabolic balance [103]. β-catenin is a key effector of the Wnt signalling cascade, which links the key transcription factors like Pdx1a and MafA to uphold β-cell identity and insulin biosynthesis. Pdx1a is a regulatory driver of β-cell differentiation and maintaining production of functional insulin. DNA hypermethylation of Pdx1a can epigenetically silence its expression and impair insulin secretion, and promote β-cell dysfunction and T2DM development [107]. MafA is another critical transcription factor for insulin production, and its expression is governed by Wnt/ β-catenin signalling, which stimulates MafA transcription and ensures adequate insulin synthesis in response to glucose stimulation. Disruption of Wnt/ β-catenin signalling compromises this regulatory mechanism and causes β-cells dedifferentiation, which in turn results in a shift in gene expression patterns that mimic immature or non-insulin producing endocrine cells. Depletion of the β-cell identity negatively affects insulin secretion and enhances disease severity [108].

## Wnt signalling and epigenetic regulation

The Wnt/ β-catenin signalling cascade controls gene expression through epigenetic mechanisms by influencing the activity of key metabolic genes, which are induced in the presence of incretin and insulin signalling. Both histones and DNA modification are crucial for modulating transcription of the insulin gene and genes responsible for β-cell proliferation and survival. β-catenin enhances regulation of these target genes by recruiting HATs which remodel chromatin structure and maintain precise control over insulin gene expression, thereby staining normal β-cell function and glucose homeostasis. This regulation is crucial for the delicate balance between β-cell renewal and apoptosis in order to preserve pancreatic function and metabolic homeostasis [109].

A recent study of heterozygous miR-26a transgenic mice revealed decreased Ten-Eleven Translocation Methylcytosine Dioxygenase 2 (TET2) expression throughout postnatal islet cell differentiation and increased postnatal islet cell populations. Beyond DNA methylation, targeted modulation of histone acetylation also impacts β-cell abundance. HDAC5 and HDAC9 deficiency has been associated with enhanced β -cell generation, while overexpression of HDAC4 and HDAC5 suppresses β-cell formation in ex vivo pancreatic models [110].

## Wnt/β-catenin and GLP-1 receptor expression

Emerging evidence highlights the role of Wnt/ β -catenin signalling in governing incretin responsiveness through epigenetic control of GLP-1 receptor (GLP-1R) expression. Hypermethylation of CpG sites within the GLP-1R promoter region suppresses its transcription, leading to diminished receptor expression and impaired GLP1-R-mediated insulin secretion. Conversely, activation of Wnt signalling mitigates this epigenetic repression by upregulating GLP1-R transcription, thereby preserving β-cell sensitivity to incretin hormones and enhancing glucose-stimulated insulin release [111].

Chen et al. (2024) highlighted the multifaceted role of Wnt/ β-catenin signalling in orchestrating bone metabolism, systems inflammation, and glucose homeostasis especially in relation to T2DM [112]. Diabetic states impair skeletal homeostasis; however, β-catenin, a principal mediator of Wnt signalling, restores osteogenic potential by directing the differentiation of bone marrow-derived mesenchymal stem cells (BMSCs) into osteoblasts while repressing adipogenesis. Its activation through the LRP5/6/GSK-3β axis stimulates bone-specific gene expression and fosters skeletal regeneration, mitigating the bone turnover deficits associated with T2DM [113]. By enhancing β-catenin signalling, GLP1R agonists such as exenatide support bone remodelling through the activation of osteogenic pathways, inhibition of bone resorption and suppression of NF-κB- associated inflammatory responses [113]. Exenatide offers distinct skeletal benefits over insulin by more potently activating β-catenin signalling, which supports bone formation and limit inflammation. Importantly β-catenin also antagonises the inhibitory actions of DKK1 and sclerostin, which are key antagonists of Wnt signalling that contribute to skeletal/bony impairment in diabetes [114]. In the context of diabetes, exenatide modulates β-TrCP, a key component of the β-catenin destruction complex, thereby reducing oxidative stress and inflammatory responses [115]. The collective actions of Wnt/ β-catenin activation, NF-κB inhibition, and suppression of adipogenesis underscore the therapeutic potential of targeting the β-catenin pathway to improve bone health and mitigate systemic inflammation in T2DM. Collectively, these findings highlight β-catenin signalling as a foundational axis in the design of next-generation therapeutics for diabetic bone and metabolic disorders.

## GLP-1 and its epigenetic regulation in T2DM

### GLP-1 Biology and signalling pathway

Glucagon-like peptide-1 (GLP-1), an incretin hormone produced via proteolytic processing of proglucagon, is primarily secreted by L-cells in the distal intestine, with secondary expression in pancreatic alpha-cells and the brainstems nucleus of the solitary tract [116]. GLP-1 is a key regulator of glucose homeostasis, exerting its effects by stimulating insulin secretion, suppressing glucagon release and modulating appetite and satiety [117].

GLP-1 exerts its metabolic functions thought the GLP-1R which is a G protein-coupled receptor that initiates a series of intracellular signalling events [118]. This activation leads to the stimulation of adenylyl cyclase and a subsequent rise in cAMP, which in turn activates downstream effectors such as PKA and EPAC [119]. Together these pathways facilitate the amplification of insulin secretion in response to elevated glucose levels. In parallel, activation of the GLP1R engages the phosphoinositide 3-Kinase (P13K)/AkT pathway, thereby enhancing β-cell survival, promoting insulin sensitivity and cellular proliferation [120]. GLP-1 also modulates intracellular calcium levels which is a critical mediator of insulin granule exocytosis. Through these coordinated mechanisms GLP-1 plays an essential role in optimising insulin secretion and maintaining metabolic homeostasis [47]. Owing to its broad-spectrum metabolic benefits GLP-1 is a key target for T2DM therapy and GLP-1 Ras are widely used to improve metabolic outcomes and promote weight loss.

### Epigenetic regulation of GLP-1 pathway

Emerging research underscores the role of epigenetic modifications in regulating GLP-1R expression, with direct implications of β-cell responsiveness to incretin-based therapies. Both DNA methylation and histone modifications modulate the transcriptional activity of GLP-1R, thereby influencing GLP-1 mediated insulin secretion and overall metabolic homeostasis. Hypermethylation of CpG islands within the GLP-1R promoter has been associated with decreased receptor expression and impaired GLP-1 mediated insulin secretion contributing to β-cell dysfunction [51]. This suggests that hypermethylation lowers incretin sensitivity and may partly explain the suboptimal therapeutic response to GLP-1 based treatments observed in a subset of individuals with T2DM.

In addition to DNA methylation, histone modifications critically influence chromatin architecture, thereby dictating the transcriptional accessibility of genes involved in GLP-1 signalling. These modifications determine whether the pathway remains transcriptionally active or silenced. Moreover GLP-1 itself modulates the β-cell epigenetic landscape by regulating the expression of key transcription factors such as Pdx1 and MafA, which are indispensable for insulin synthesis and β-cell viability [121]. These epigenetic adaptations serve as protective mechanisms that help preserve β-cell function and slow the progression of T2DM. As such, targeting epigenetic regulators offers a promising therapeutic strategy to enhance GLP-1R signalling [122]. Thus, by understanding the epigenetic regulation of the GLP-1 pathway researchers are exploring novel strategies to enhance incretin-based therapies [123]. Specifically reversing DNA hypermethylation or remodelling histone marks at the GLP-1R promoter may augment the efficacy of GLP-1R agonists and restore β-cell responsiveness in individuals with reduced incretin sensitivity.

## GLP-1 receptor agonists (GLP-1RAs) in T2DM therapy

### Classification of GLP-1RAs

GLP1-R agonists are categorised according to the duration of their action. This factor significantly impacts both therapeutic outcomes and patient compliance [50]. Short acting GLP1-RAs such as exenatide (administered twice daily) and lixisenatide are predominantly effective in managing postprandial glycaemic control. Their mechanism involves delaying gastric emptying and suppressing glucagon secretion thereby reducing sharp spikes in blood glucose after meals. Nevertheless, the frequent administration poses a barrier to adherence thereby limiting their clinical use [124, 125]. Contrarily long-acting GLP-1RAs such as liraglutide (once daily), dulaglutide and semaglutide (once weekly) offer sustained glycaemic control due to their prolonged receptor activation. These agents also provide greater weight loss benefits and have better patient compliance due to their less frequent dosing regimen [126–128]. Notably, semaglutide has exhibited superior efficacy in both glycaemic control and weight management, positioning it as a leading therapeutic option for management of T2DM and obesity [51].

### Mechanisms of GLP-1RAs

GLP-1R agonists replicate the physiological actions of GLP-1 thus contributing to improved glycaemic control, metabolic homeostasis and cardiovascular health [117]. Their therapeutic efficacy is mediated through a range of complementary mechanisms. Primarily, they enhance glucose-dependant insulin secretion from pancreatic β-cells ensuring appropriately matched insulin to hyperglycaemic states and thereby attenuating postprandial hyperglycaemia [116]. Concurrently, they suppress glucagon secretion from pancreatic alpha cells bringing about a reduction in hepatic glucose production and triggering a lower fasting glucose levels [118].

In addition to their role in glucoregulation, GLP-1R agonists slow gastric emptying thereby delaying nutrient absorption and attenuating postprandial glucose spikes which stabilise blood glucose levels. These agents also modulate central appetite pathways by acting on hypothalamic neurons to promote satiety thus reducing calorie intake and leading to significant weight loss [129]. Moreover GLP-1RA’s exert cytoprotective effects on pancreatic β-cells by inhibiting apoptosis, promoting cell survival and sustaining insulin secretory capacity over time [130]. This β-cell protective effect is particularly advantageous in the long-term management of T2DM as it slows down disease progression and mitigates β-cell exhaustion.

### Clinical benefits of GLP-1Ras

GLP-1RAs offer a broad spectrum of clinical benefits that extend beyond glycaemic regulation [51]. These agents have been shown to significantly reduce Haemoglobin A1c (HbA1C) levels by approximately 1–2% with minimal risk of hypoglycaemia. This makes them a safer alternative to insulin or sulfonylureas particularly for patients with preserved β-cell function [131]. As effective weight management therapies, GLP-1RAs can induce body weight reduction of approximately 3–7% in T2DM trials, with greater reductions (5–15%) reported for high dose semaglutide in randomised clinical trials. The observed weight loss is primarily attributed to their central appetite-suppressing actions, thus reducing calorie intake and enhancing overall metabolic profile [132]. Notably Liraglutide and Semaglutide have shown cardiovascular benefits by significantly lowering the incidence of major adverse cardiovascular events (MACE), including myocardial infarction and stroke [130]. These cardioprotective properties have led major international guidelines to recommend GLP-1 RAs as preferred agents for T2DM patients, who have high-risk of cardiovascular disease [126]. These disease-modifying characteristics position GLP-1RAs as efficient agents in the early intervention and long-term management of T2DM.

### Limitations and side effects

The therapeutic profile of GLP-1Ras is counterbalanced by several limitations that warrant careful consideration. Commonly gastrointestinal side effects such as nausea, vomiting, diarrhoea and constipation are reported particularly in the early stages of treatment. Although these symptoms are often short lived, they can negatively impact patient adherence and overall treatment satisfaction [133]. More serious, albeit rare, adverse events include pancreatitis and gallbladder disease. While a systemic review [134] has suggested an increased risk of gallbladder and biliary disorders with GLP-1RA use, large-scale observational studies have not established a significant association with pancreatitis [135]. In animal studies GLP-1Ras have been associated with thyroid C-cell hyperplasia and medullary thyroid carcinoma in rodent models. However, these findings have not been replicated in human clinical trials and epidemiological studies, and current evidence does not support a causal relationship in humans [136]. Another significant barrier is cost, particularly, newer agents, like Semaglutide, can be expensive depending on country, healthcare system, insurance coverage and formulations. In some settings monthly costs may exceed $1000 USD. This financial burden poses challenges to long-term accessibility and raises concerns about the broader implementation of these therapies in routine care [137].

## Conclusion: epigenetics as a target for T2DM therapy

This review highlights the profound role of epigenetics in the pathogenesis and therapeutic landscape of T2DM. The intricate interplay between DNA methylation, histone modifications and noncoding RNAs governs fundamental processes such as β -cell function, insulin resistance and chronic inflammation thence driving disease onset and progression. Notably, the heritable yet reversible nature of epigenetics alterations presents a promising framework of precision medicine. This bridges gap between genetic predisposition and environmental exposures. Alongside improved insights into disease mechanisms the potential to treat T2DM by targeting epigenetic changes is particularly promising. Emerging interventions such as DNMT inhibitors, HDAC inhibitors, and miRNA-based therapies suggest a paradigm shift from symptomatic management to molecular-level interventions that address epigenetic dysregulation rather than merely controlling hyperglycaemia. The pleiotropic effects of GLP-1RAs on metabolic pathways further underscore their potential as part of a comprehensive therapeutic strategy that addresses both metabolic dysfunction and epigenetic regulation. Nevertheless, the precise epigenetics effects of GLP-1RAs remain to be fully elucidated, warranting further investigation to clarify their role within this expanding therapeutic paradigm.

One of the most compelling insights emerging from epigenetic research is the concept of transgenerational epigenetic inheritance and the understanding that lifestyle, nutrition and metabolic health can imprint lasting molecular changes that influence the health of future generations. This perspective shifts diabetes prevention from an individual-focussed approach to a broader intergenerational model that emphasizes the need for early intervention strategies that not only treat the disease but also mitigate its epigenetic transmission. Despite considerable progress key questions remain unresolved. The long-term stability of epigenetic modifications, their utility as predictive biomarkers, and the extent to which environmental exposures shape inherited epigenetic patterns require deeper investigation. Integrating epigenetic profiling into clinical practice will be essential for developing more precise and targeted therapies that address the molecular underpinnings of T2DM. Ultimately epigenetic regulation offers a transformative lens through which understanding of diabetes extends beyond static genetic predisposition. As research advances, therapies targeting epigenetic dysregulation may redefine diabetes care, shifting focus from symptom control to molecular level prevention and potential reversal of disease progression.

## Data Availability

No datasets were generated or analysed during the current study.
